# The Performance of a Spherical-tip Catheter for Stent Post-dilation: Finite Element Analysis and Experiments

**DOI:** 10.3389/fphys.2021.734565

**Published:** 2021-08-31

**Authors:** Lin Qi, Wenbo Zhu, Wei Qian, Lisheng Xu, Ying He, Feihu Zhao

**Affiliations:** ^1^College of Medicine and Biological Information Engineering, Northeastern University, Shenyang, China; ^2^Engineering Research Center of Medical Imaging and Intelligent Analysis, Ministry of Education, Northeastern University, Shenyang, China; ^3^Key Laboratory of Medical Image Computing, Ministry of Education, Northeastern University, Shenyang, China; ^4^School of Control Engineering, Northeastern University at Qinhuangdao, Qinhuangdao, China; ^5^School of Energy and Power Engineering, Dalian University of Technology, Dalian, China; ^6^Department of Biomedical Engineering, Eindhoven University of Technology, Eindhoven, Netherlands; ^7^Department of Biomedical Engineering, Zienkiewicz Centre for Computational Engineering, Faculty of Science and Engineering, Swansea University, Swansea, United Kingdom

**Keywords:** post-dilation, spherical-tip catheter, finite element analysis, percutaneous coronary intervention, finite element model

## Abstract

At present, percutaneous coronary intervention (PCI) is the most effective treatment of coronary artery stenosis. However, in case post-dilation of the stent is needed, the tip of the commonly used post-dilation balloon catheter cannot always pass through the stent smoothly, especially when it is situated in the curved part of the vessel. To improve the performance of traditional post-dilation balloon catheter, a preliminary design of a novel catheter with a spherical-tip is proposed. Since the performance of this spherical-tip catheter is still unclear, in this study, finite element analysis (FEA) and experimental validation of blood vessel with different curvature radii were performed to test and evaluate the performance of the spherical-tip catheter design. The comparative results between the two types of catheters demonstrate that in the simulated post-dilation process, the spherical-tip catheter is easier to pass through the stent placed in the curved vessel without the deformation of the stent strut, and can theoretically reduce the operation time and improve the safety of the operation. Furthermore, the strong consistency between simulation and experiment indicates that the finite element (FE) model can be a helpful tool for future optimization and evaluation of novel catheters, so as to save time and budget in product development and reduce/replace animal studies.

## Introduction

In recent years, the incidence and mortality of coronary heart disease (CHD) have increased significantly (Gagnier et al., 2010; Liu et al., 2012). It is estimated that by 2020, the death rate of coronary heart disease will increase by 50% (approximately 25 million people per year) (Anderson et al., 2016). As percutaneous coronary intervention (PCI) is used primarily for opening a blocked coronary artery and restoring arterial blood flow to heart tissue, and without requiring open-heart surgery, it has the advantages of a short course of treatment, low trauma, and an obviously curative effect, which makes it the most effective treatment of CHD (Williams et al., 2000; Stone et al., 2010).

**Figure 1:**
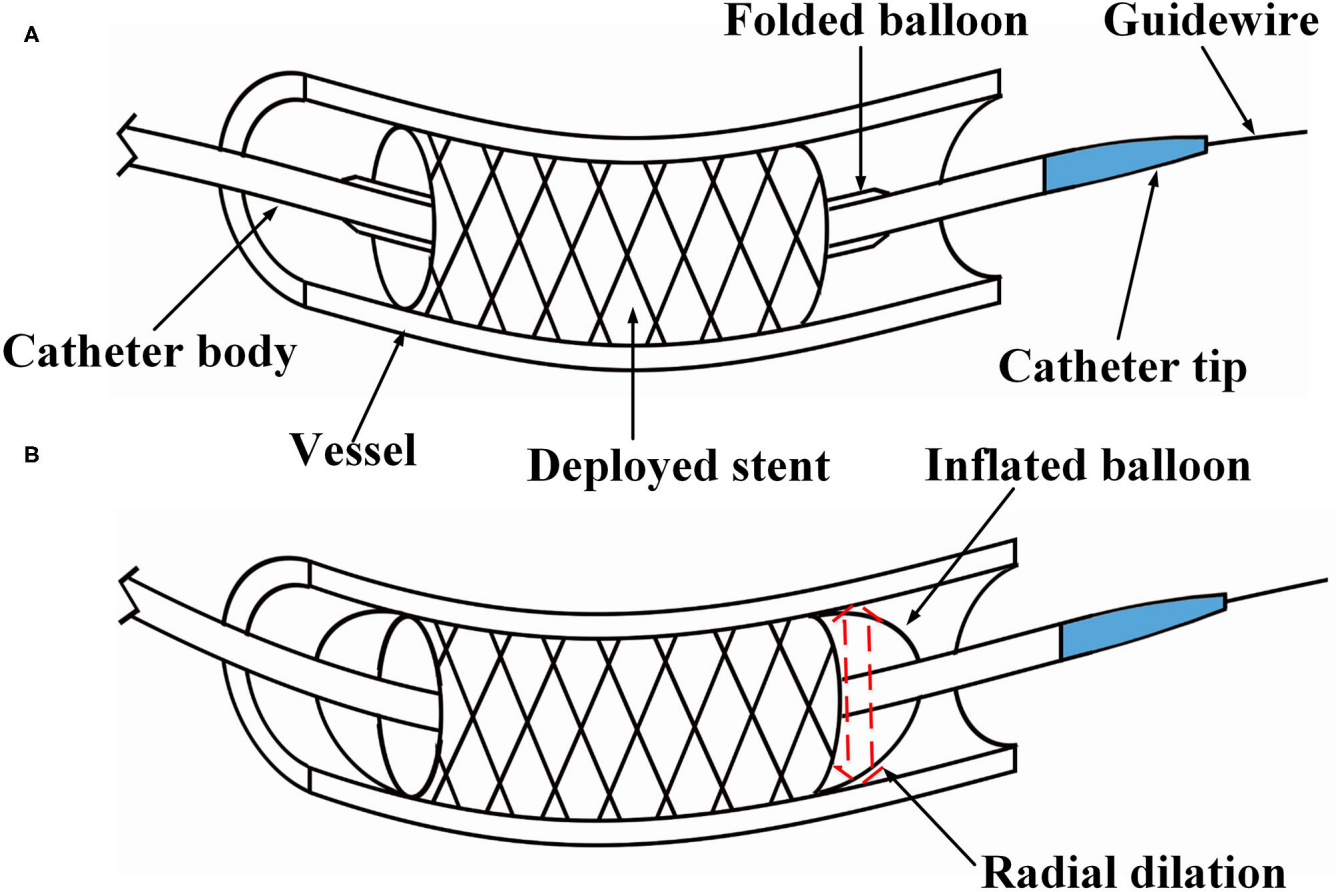
Illustration of percutaneous coronary intervention. **(A)** The stent is placed at the narrow section of the vessel, and **(B)** under in-stent dilation during the post-dilation procedure.

In clinical practice, balloon pre-dilation is often performed before stent implantation (Migliorini et al., 2010), as it reduces the stenosis of lesions and herewith reduces the damage of stent coating, which is caused during placement. The tip of the pre-dilation catheter has a conical shape, which is conducive for the catheter passing through narrow lesion sites (see Figure 1A). Post-dilation refers to in-stent dilation of the balloon after stent deployed, applying a higher pressure than that used during stent implantation. This operation is performed to ensure the complete attachment between the stent and the vascular wall, which reduces the probability of in-stent thrombosis and the rate of restenosis after the operation (see Figure 1B). Balloon post-dilation has also been developed as an option to reduce the degree of aortic regurgitation (Nombela-Franco et al., 2012), and there is no stenosis in the pathway of the post-dilation catheter.

**Figure 2:**
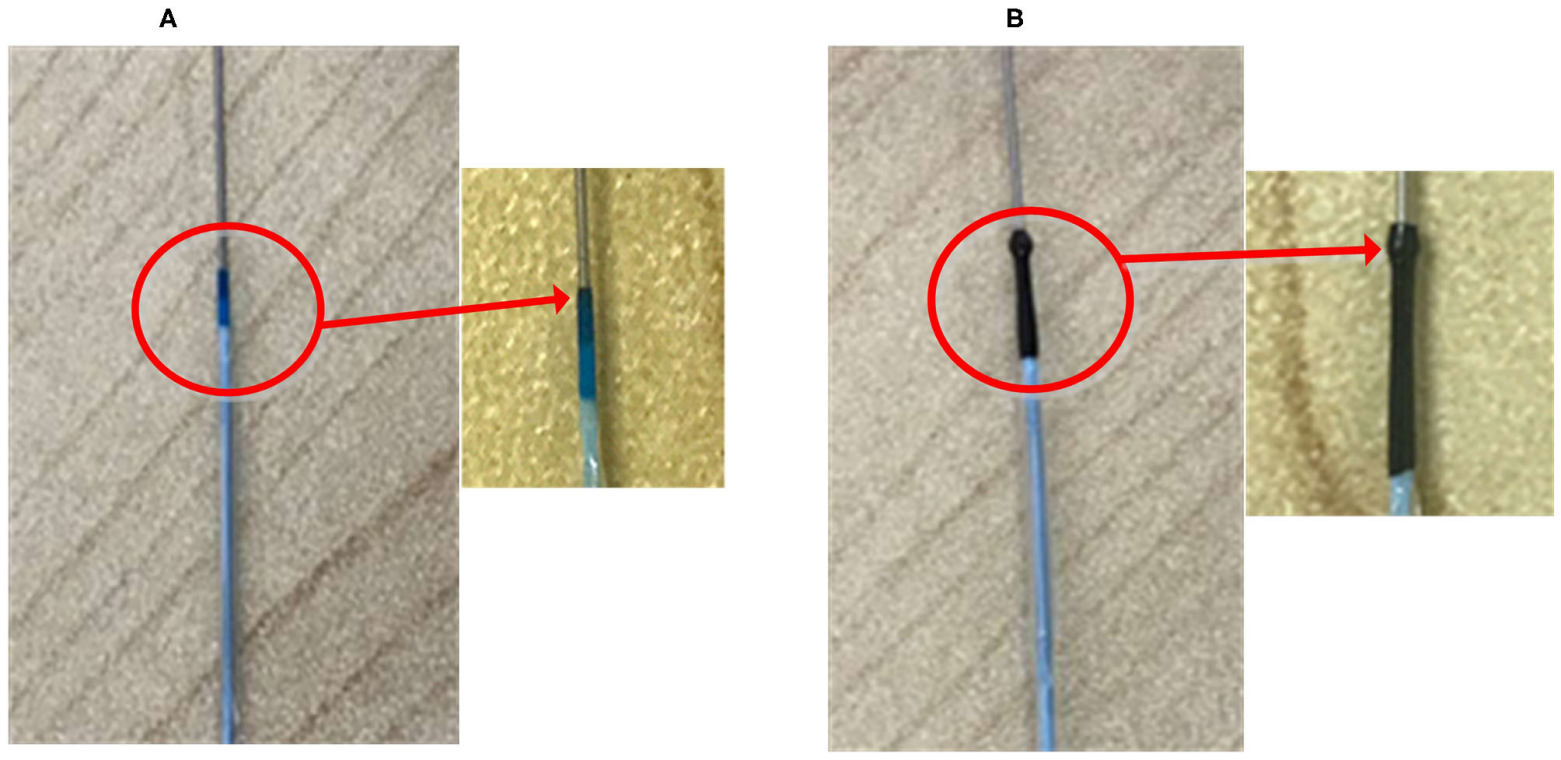
Two types of PCI catheters. **(A)** Traditional balloon catheter and **(B)** spherical-tip balloon catheter.

However, since the tip of the most commonly used post-dilation balloon catheter in the clinic has a conical structure (see Figure 2A), the catheter tip can easily get stuck in the stent (Colombo and Stankovic, 2007; Watanabe et al., 2013). In particular, when the catheter has to pass a curved vessel with an implanted stent that consists of rectangular struts, it is difficult for the catheter to pass smoothly through the stent. Sometimes, further advancement of the catheter becomes impossible as it is fully stuck in the stent, and the surgeon has to move the catheter back and forth to find another way through. The passability of post-dilation catheter, however, may cause large stent deformation and damage to the vessel wall, not unlikely leading to acute myocardial infarction and in unfortunate cases even induce death of the patient. Therefore, Sun (Sun, 2018) proposed a novel preliminary design of a balloon catheter with a spherical-tip (see Figure 2B). Before clinical trials can be carried out, the evaluation on the performance of this new spherical-tip catheter design is necessary, especially on the passability through the dilated stent.

The primary factor that affects the catheter passability in post-dilation is the contact behavior between the catheter tip and the stent strut. The force loaded on the tip due to the blood flow will be much lower than the contact force between the catheter tip and strut under physiological conditions. The influence of blood flow is ignored in this study in order to simplify the model. If a special intravascular catheter is employed, such as a catheter with a fiber-optic probe for intra-vascular sensing, the effect of bulk blood flow on the catheter tip should be taken into account (Ghata et al., 2015). Lawton et al. (2000) presented a physical model and a numerical algorithm to simulate the insertion and navigation of a catheter into an arterial system, and the influence of blood flow was also ignored in their model. By contrast, this study aims to demonstrate the better passability of the novel catheter by comparing it to a commonly used one in curved vessel, and a reasonable simplification was made.

Previous studies have demonstrated the feasibility of using finite element analysis (FEA) to validate/evaluate the performance of newly developed PCI procedures. For example, FEA was used to predict whether two different types of stents provoke different levels of stress in the vascular wall (Lally et al., 2005). On the one hand, other researchers used FEA to numerically evaluate commercial stents, which were deployed in straight or curved arteries with plaque (Jung and Kim, 2016). On the other hand, stent thrombosis is a frequently met problems after stenting. Chesnutt and Han (2015) utilized a discrete element method to simulate the interactions of fluid and platelets and red blood cells around struts. It is found that most previous studies focused on the implantation of the stent or the problems after stenting. To the best of our knowledge, the PCI post-dilation catheter delivering problems, regarding advancement through a deployed stent, has not gained much attention in the numerical investigation of PCI procedures.

Therefore, in this study, the performances of a proposed spherical-tip catheter and a traditional catheter were further demonstrated by a combination of FEA and experimental verification. In both FEA and experimental measurement, the contact force between the catheter tip and stent surface is used for characterizing the passability of the catheters. It is anticipated that the outcome of the analysis could provide important implications for future animal experiments and clinical trials. Meanwhile, the experimental verification of the FE model should give confidence that FEA can be used as a tool for future optimization and redesign of the catheter.

## Materials and Methods

In this section, there are two main steps to the methodology: (1) A simplified FE model was created and verified through specifically designed experiments, and (2) the FE modeling was then applied to a more complicated catheter-stent-vessel configuration.

###  Simplified Finite Element Model

**Figure 3:**
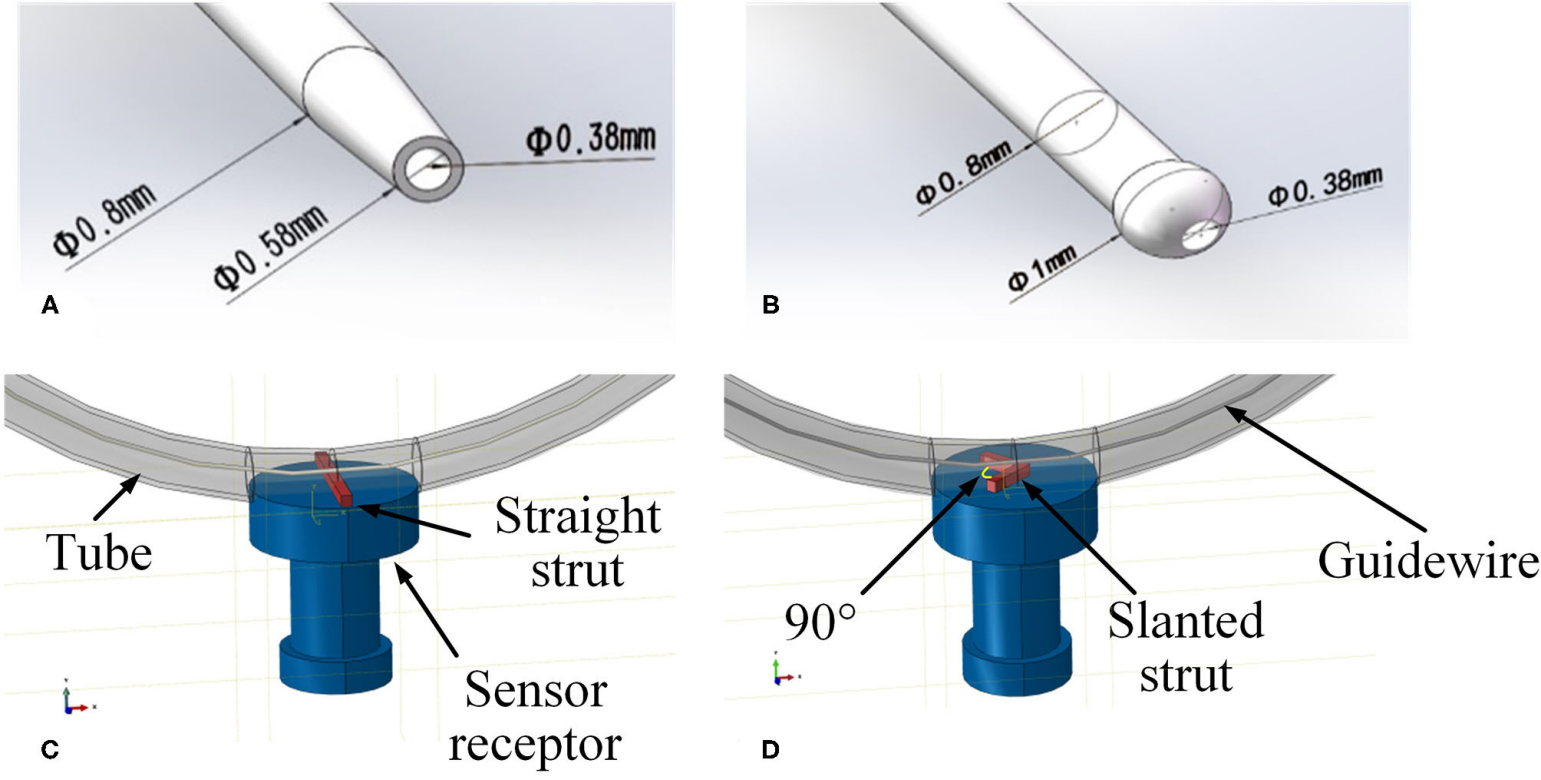
Geometries of experimental FE model. **(A)** Tip of the traditional catheter, **(B)** tip of the spherical-tip catheter, **(C)** straight strut model, and **(D)** slanted strut for finite element model.

**Figure 4:**
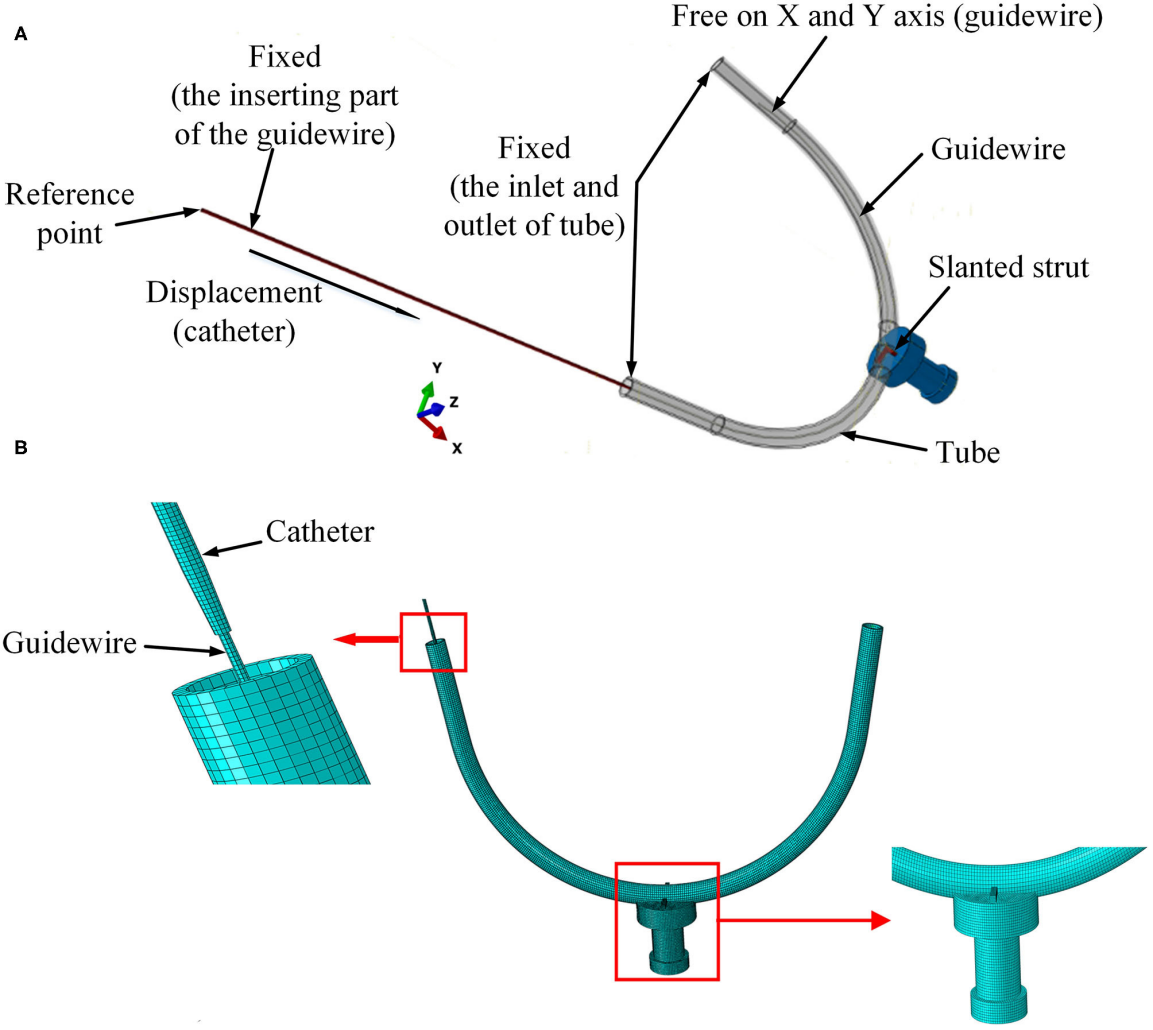
Setup of experimental FE model. **(A)** Boundary conditions for the FE model and **(B)** mesh of the 3D FE model.

In order to verify the feasibility and effectiveness of the FM method, a simplified FE model was constructed. The geometry of different parts, including a curved tube, a guidewire, a sensor receptor, struts of the stent, and catheters, was created in CAD software SOLIDWORKS^Ⓡ^ (Dassault Systems SolidWorks Corp., France) based on the experimental setup in the next subsection. A straight stent strut and a slanted stent strut were compared in this model because the catheter may contact the different parts of the stent. Both the straight strut and the slanted strut were fixed individually on the sensor receptor to mimic the stent struts. The cross-section of the stent strut was 1 × 1 mm. The angle between the two wings of the v-shaped slanted strut was 90°. The tube was modeled as a hollow cylinder with an inner diameter of 3 mm and a thickness of 1 mm, which was similar to the size of the normal artery (Karimi et al., 2013). According to the experimental setup in the next subsection, the radius of curvature of the tube was set to 10 mm. At the location of the sensor receptor, the tube was opened at one side and flush mounted to the sensor receptor (see Figures 3, 4 for more details). The diameter of the guidewire was 0.36 mm (Hara et al., 2002; Tanaka et al., 2009). The structure and dimension of the two catheter tips were shown in Figures 3A,B. Then, the solid model was directly imported into the CAE software ABAQUS^Ⓡ^ V6.13 (Dassault Systems Simulia Corp., France). The catheter tip was made of polyurethane (Vulkollan^Ⓡ^), while the catheter body was made of polyamide (Nylon^Ⓡ^) (Hunan APT Medical Inc., China). The material of the sensor receptor was stainless steel. The struts were made of iron with the mechanical properties listed in Table 1.

**Table 1:** Properties of the materials in the experimental finite element (FE) model.

	**Young's modulus (MPa)**	**Poisson's ratio**
Strut (Wu and Wei, 1991)	150,000	0.3
Catheter tip (Stoimenov et al., 2013)	380	0.4
Catheter body (Stoimenov et al., 2013)	2,900	0.4
Guidewire (Wei et al., 2015)	30,000	0.3
Curved tube (Tsukruk et al., 2015)	7.8	0.47
Receptor (Wu and Wei, 1991)	200,000	0.3

As shown in Figure 4A, the strut and the sensor receptor were assumed to be tied together, which meant there was no displacement on the interface between strut and sensor. The curved tube was fixed at both its ends (inlet and outlet). The strut was fixed at the interface with the sensor, which itself was also fixed. The inserting catheter part of the guidewire was fixed. The rotations on the X-Y-Z axis and displacement on the Z axis of the other side of the guidewire were restricted. The catheter was displaced at its tail with constant speed of 30 mm/s. For simplicity, the FE contact analysis used the frictionless general contact method in Abaqus/Explicit.

Linear elastic solid elements [type 8-node brick “reduced-integration” elements(C3D8R)] were selected for the FM mesh of each part in the model. The straight strut was meshed with 1,750 elements and 2,556 nodes. The slanted strut was meshed with 1,836 elements and 2,541 nodes. The spherical-tip catheter was discretized by 8,448 elements and 16,524 nodes. The traditional catheter was discretized by 9,785 elements and 14,964 nodes. The vessel was discretized by 10,460 elements and 21,460 nodes. The guidewire was meshed with 5,400 elements and 12,159 nodes. The sensor receptor was meshed with 32,375 elements and 35,895 nodes. The mesh of the 3D FE model was shown in Figure 4B.

###  Finite Element Analysis Validation by Conducting Experiment

**Figure 5:**
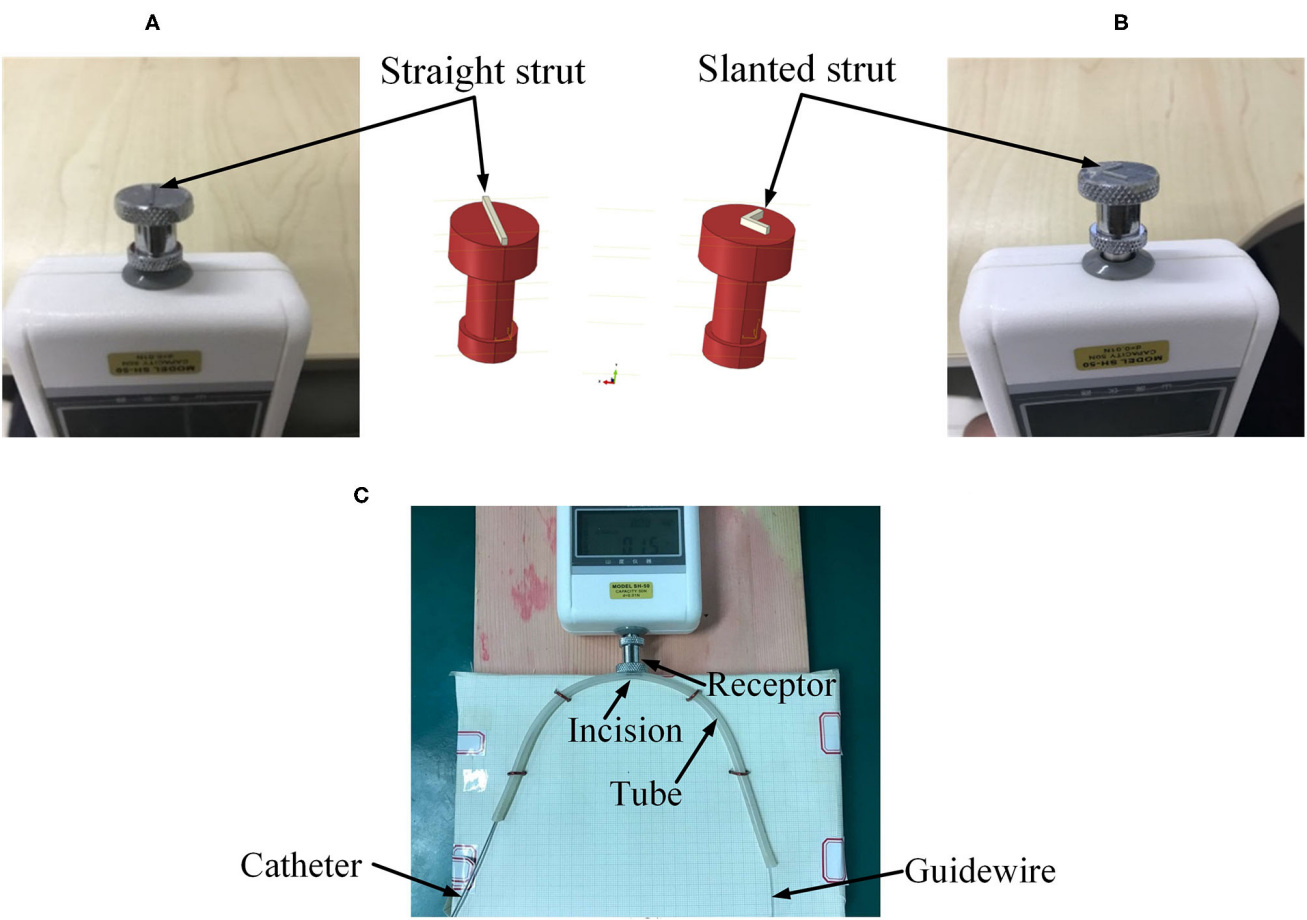
Experimental setup. **(A)** Straight strut, **(B)** slanted strut, and **(C)** assembled experimental model.

The accuracy of the FEA result was assessed by conducting an experiment to measure the contact forces between the catheter tip and the stent strut. A rubber tube was used to mimic the blood vessel. The resolution of the force sensor (SH-50, Sundoo Instruments Co., Ltd., China) is 0.01 N. As shown in Figure 5C, the sensor was fixed on a board, while the tube was fixed on a scale paper. The tested tube used in this study had a curvature radius of 10 mm. An incision was made at the outer wall in the middle of the tube. The length of the incision was equal to the diameter of the sensor receptor. A straight strut and a slanted strut were set individually on the sensor receptor to mimic the stent struts as shown in Figures 5A,B. The proximal side of the guidewire was fixed. Afterward, the manipulator inserted the catheter along the guidewire with the constant speed of 30 mm/s. Once the catheter was in contact with the strut, the contact force between the sensor and the strut was recorded. The contact force for each catheter was repeatedly measured 10 times.

### Finite Element Analysis for Post-dilation

**Figure 6:**
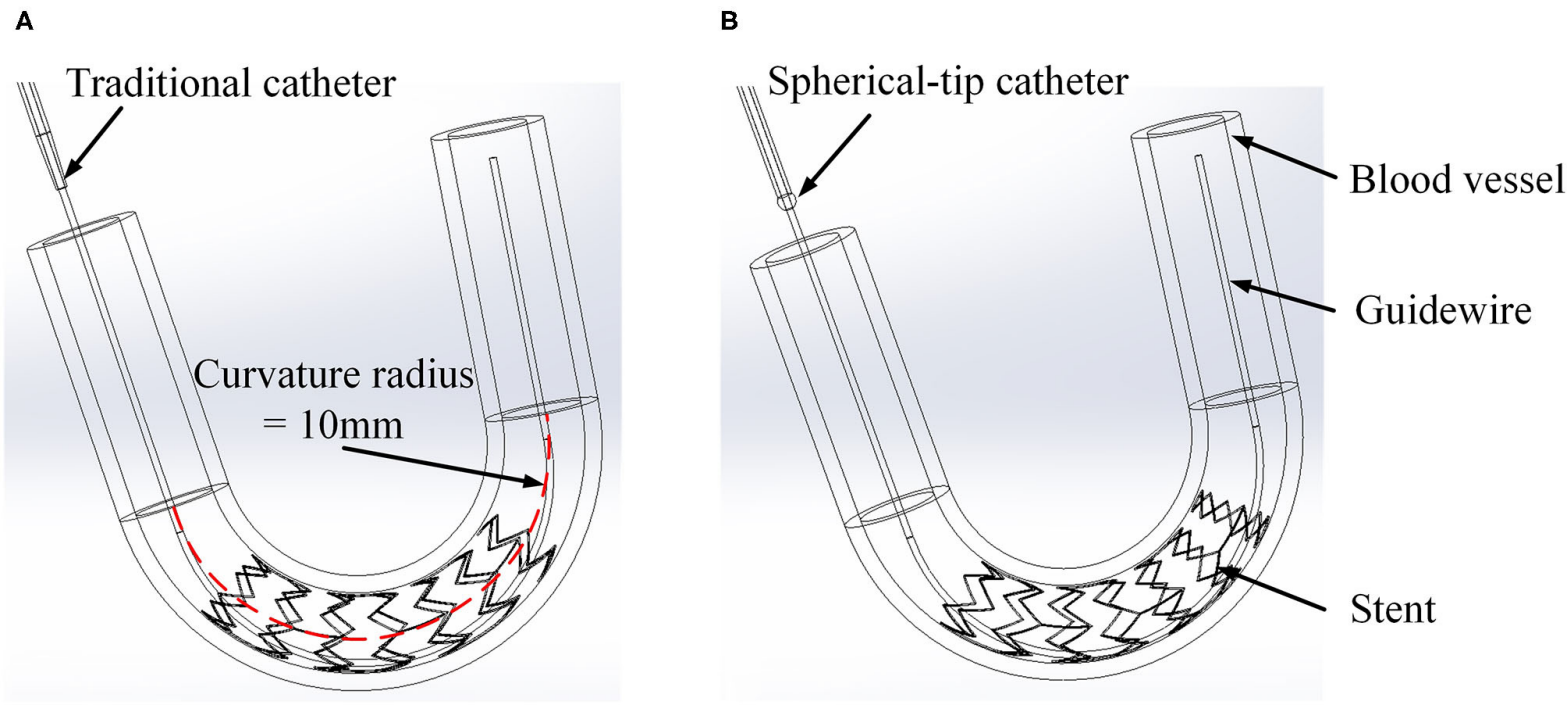
Three-dimensional FE models of catheter, stent, and vessel. **(A)** Traditional catheter model and **(B)** spherical-tip catheter model.

**Figure 7:**
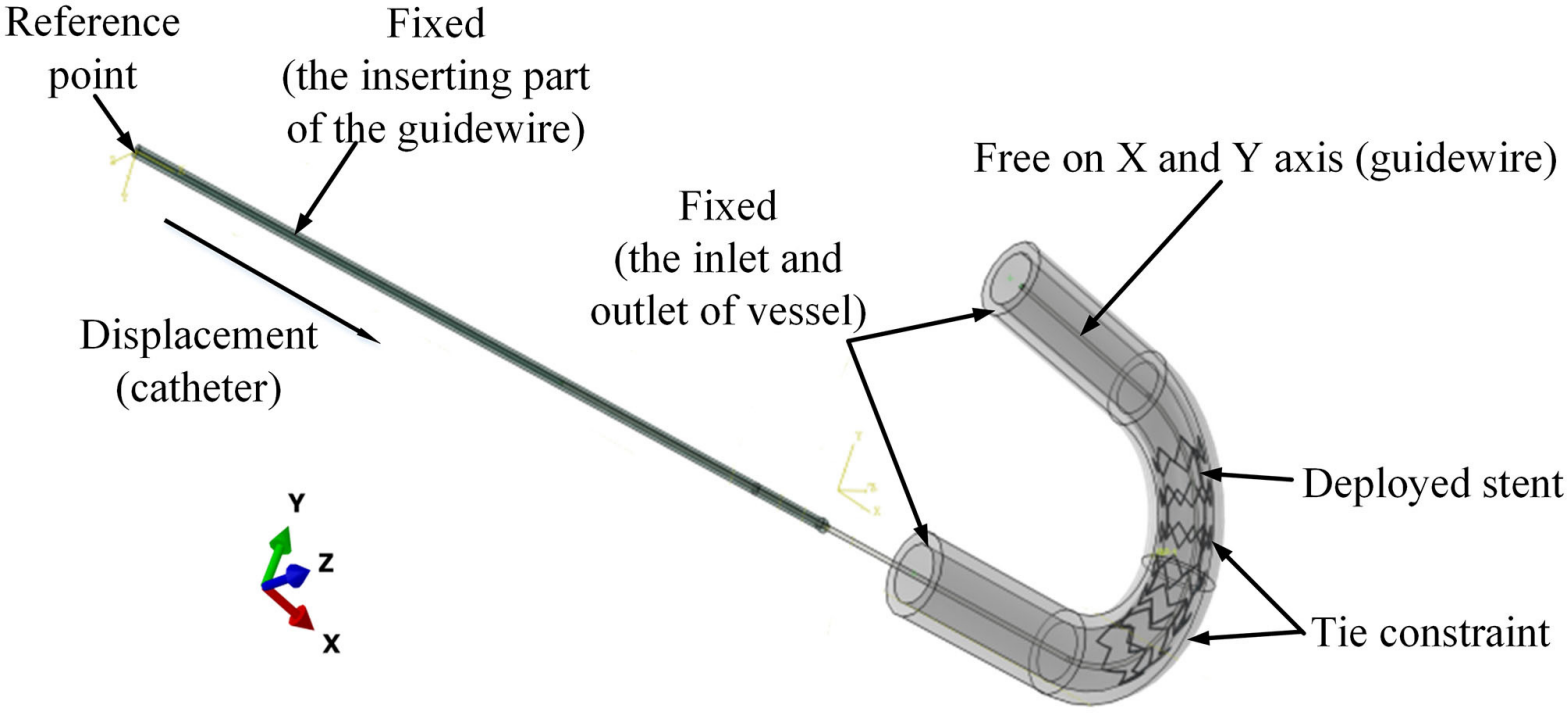
Boundary conditions for the FE model of catheter-stent-vessel configuration (with curvature radius of 10 mm).

The preliminary work had provided a basis for the following simulation. In the post-dilation FEA model, there were four parts: stent, catheter, guidewire, and blood vessel. The blood vessel was modeled as a hollow cylinder with an inner diameter of 3 mm and a wall thickness of 1 mm (Karimi et al., 2013). The cross-section of coronary stent strut can be circular or rectangular. The coronary stent struts with different cross-section types have their own advantages. The geometry of the stent was designed according to the product provided by the manufacturer (Hunan APT Medical Inc., China), which was fabricated with rectangular struts of 0.1 × 0.1 mm rectangular cross-section. Rectangular strut stent has good supporting performance and less chance of retracting (Kim et al., 2008). The diameter of the guidewire was 0.36 mm (Hara et al., 2002; Tanaka et al., 2009). The curvature radius of the stent and vessel was set to 10 mm. The assembled four parts were presented in Figures 6A,B, and the whole FE model and boundary conditions of the model were shown in Figure 7. In addition, since the shape of the real vessel varies, in order to evaluate the performance of the spherical-tip catheter under different curved conditions, the FE simulation was further used to verify the performance of the spherical-tip catheter and the traditional one passing through the blood vessels with curvature radii of 20 mm and 30 mm.

These geometries were imported into ABAQUS v6.13 as shown in Figure 7. The material of stent was nickel-titanium alloy. The catheter tip was made of polyurethane, while the catheter body was made of polyamide. The vessel was assumed as a linearly elastic body. The arterial Young's modulus of 20–30 years old people was 0.45 ± 0.12 MPa according to the measurement results (Zhu and Chang, 1991; Holzapfel et al., 2000). All material properties are shown in Table 2.

**Table 2:** Properties of materials in the catheter-stent-vessel FE models.

	**Young's modulus (MPa)**	**Poisson's ratio**
Stent (Wang et al., 2012)	28,440	0.33
Catheter tip (Stoimenov et al., 2013)	380	0.4
Catheter body (Stoimenov et al., 2013)	2,900	0.4
Guidewire (Wei et al., 2015)	30,000	0.3
Blood vessel (Zhu and Chang, 1991)	0.45	0.3

The boundary conditions of the model are shown in Figure 7. To mimic the situation in clinical application, the displacements and rotations of two end faces of the vessel on the X-Y-Z axis were restricted. The inserting catheter part of the guidewire was fixed. The rotations on the X-Y-Z axis and displacement on the Z axis of the other side of the guidewire were restricted. The tie constraints were applied between the stent and vessel, so that there was no relative movement between the stent and vessel.

Surface-to-surface contact (Abaqus/Explicit) was applied between the outer surface of the catheter and the inner surface of the stent. In this study, the simple sliding friction model was adopted for the surface-to-surface contact as

(1) $$\begin{array}{r} F_{f}=\mu \cdot F_{N} \end{array}$$

where *F*_*f*_ is the friction force, *F*_*N*_ is the normal force, and μ is the friction coefficient with the value of 0.2 (De et al., 2008; Roszelle et al., 2014). Other contact pairs in the model used the frictionless general contact algorithm in Abaqus/Explicit. A displacement of 60 mm was imposed onto the reference point to guide the catheter through the stent. The simulation time period was set to 2 s.

The parts of the FE model were modeled with C3D8R using ABAQUS FM code. The stent was meshed with 8,892 elements and 10,670 nodes. The vessel was discretized by 33,800 elements and 44,622 nodes. The catheter was discretized by 20,320 elements and 30,096 nodes. The guidewire was discretized by 21,850 elements and 28,530 nodes.

## Results and Discussion

###  Simplified FEA and Experimental Validation

**Figure 8:**
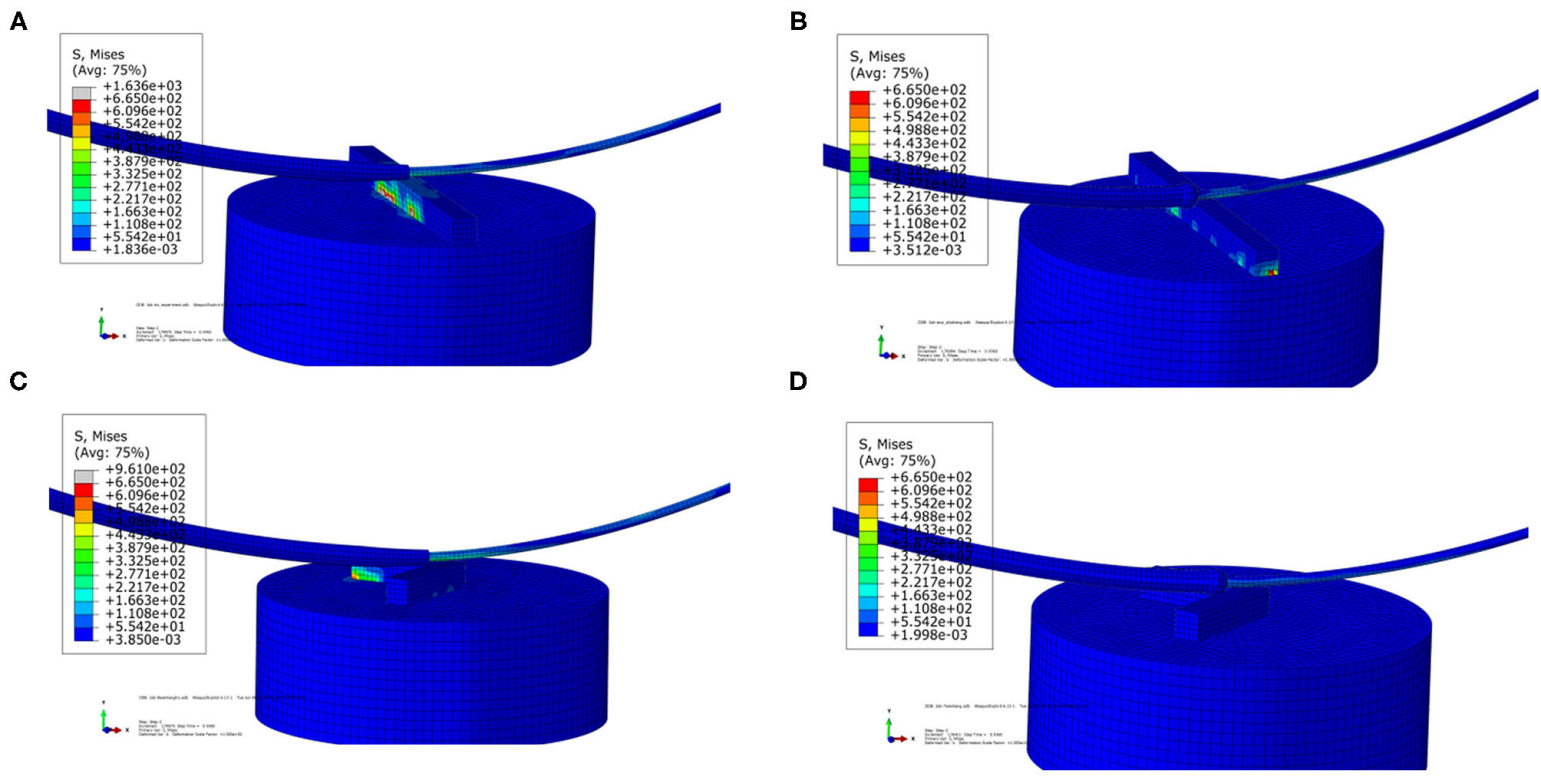
Visualizations of simulation results of the experimental FEA model. **(A)** Traditional catheter with straight strut model, **(B)** spherical-tip catheter with straight strut model, **(C)** traditional catheter with slanted strut model, and **(D)** spherical-tip catheter with slanted strut model.

In Figure 8, the stress distribution of the experimental FEA model is presented. In Figures 8A,C, the stress concentration in the strut is caused by the traditional catheter. For the spherical-tip catheter, the stress distribution is lower and more homogeneous than for the traditional catheter. Stress concentration only occurs when the catheter tips come into contact with the struts. From this perspective, the spherical-tip catheter outperformed the traditional one.

**Figure 9:**
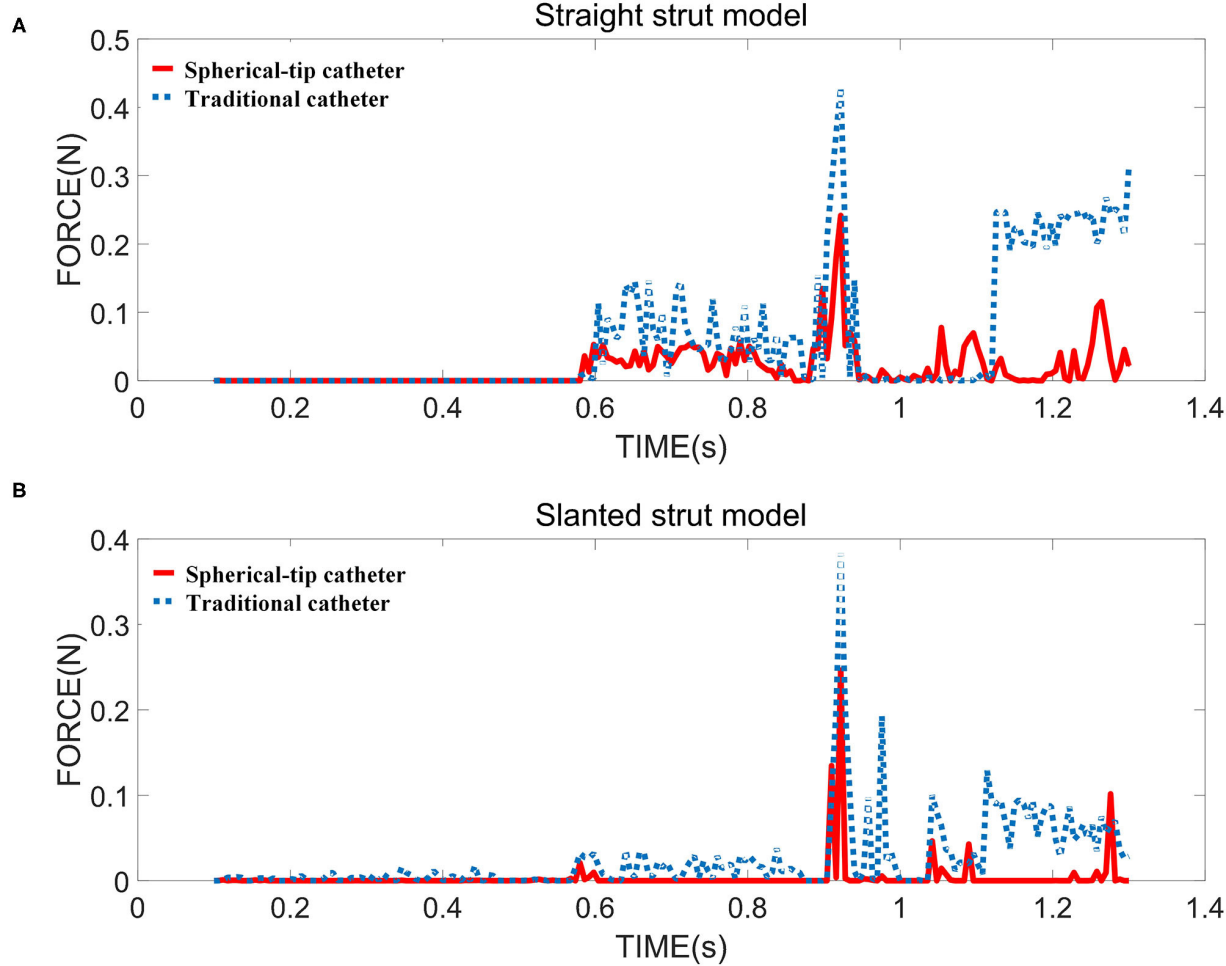
The contact force between strut and sensor for two strut models in the FE simulation i.e., **(A)** straight strut model and **(B)** slanted strut model.

Figure 9A shows the contact force between the bottom surface of the straight strut and the upper surface of the sensor receptor. Figure 9B presents the contact force for the slanted strut model under the same condition. The maximum contact force is generated when the catheter tip comes into contact with the struts. In the straight strut model, the maximum value of contact force from the traditional catheter is 0.429 N, while the maximum value of the contact force from the spherical-tip catheter is 0.242 N. In the slanted strut model, the maximum value of contact force from the traditional model is 0.383 N, while the maximum value of contact force from the spherical-tip catheter is 0.246 N. When driving those two catheters with the same conditions, the contact force between the traditional catheter tip and the stent is larger than that between the tip of the spherical-tip catheter and the stent. In Figure 9, the catheter tip contacts the strut at 0.92 s in both catheter models, and the contact force reached the first peak. From 0.92 to 1.11 s, the catheter body did not touch the strut. As the catheter continues to move along the guidewire, the contact force reaches the second peak when the catheter body touched the strut.

**Figure 10:**
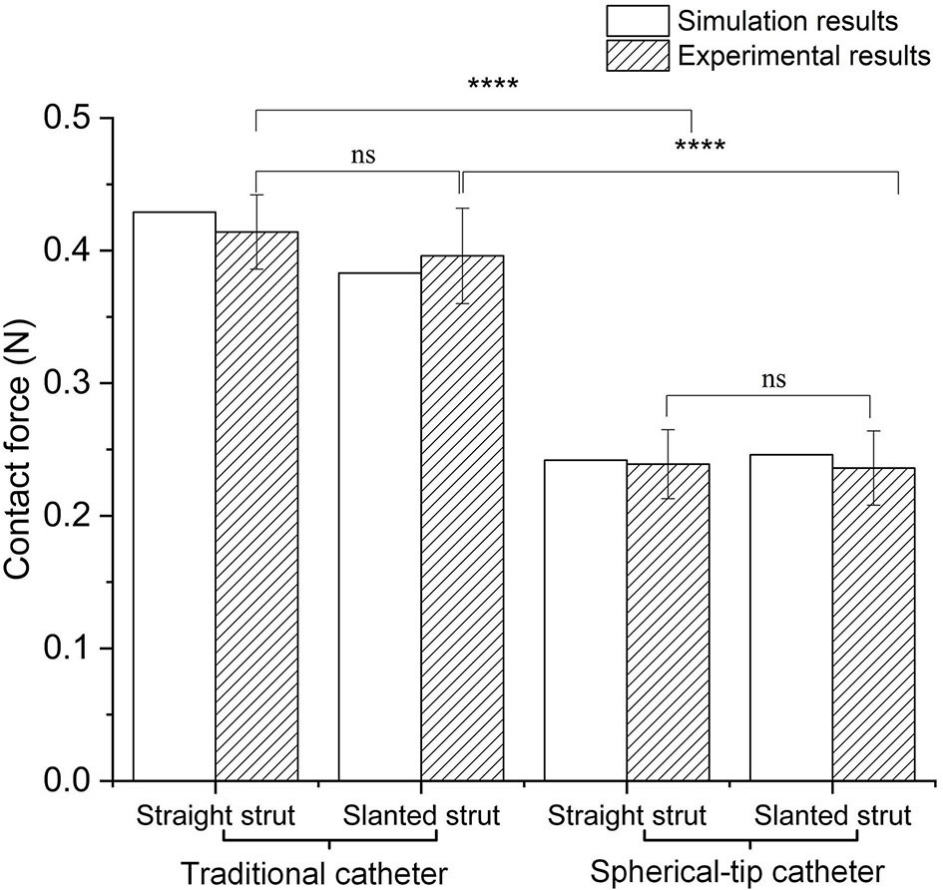
Comparison of contact force between simulation and experimental results under traditional/spherical-tip catheter groups and straight/slanted strut groups. ****p < 0.0001, ns, not significant.

In the experiments (shown in Figure 5), for the straight strut model, the measured contact force caused by traditional catheter is 0.414 ± 0.030 N (mean value ± SD calculated from 10 times measurements), while the contact force caused by spherical-tip catheter is 0.239 ± 0.027 N. For the slanted strut model, the traditional catheter has the resultant contact force of 0.396 ± 0.037 N, while the spherical-tip catheter showed the contact force of 0.236 ± 0.030 N. In addition, the experimental results were processed with *t*-test in the IBM SPSS^Ⓡ^ Statistics and are presented in Figure 10 with the simulation results. The simulation and experimental results in Figure 10 show that: (1) There is strong consistency between the simulation and experimental results; (2) the contact force between the traditional catheter tip and the strut is significantly larger than that between the tip of the spherical-tip catheter and the strut under the same conditions(*p* < 0.0001), which indicated that it was easier for the spherical-tip catheter to pass through the struts than that of traditional one; (3) for both traditional catheter and spherical-tip catheters, there is no significant difference in the contact force between straight and slanted strut groups. The work above made a preliminary evaluation of those two catheters, which had proved the feasibility of the FEA method. It sets a foundation for the following FEA simulation.

###  Results of FEA for Post-dilation

**Figure 11:**
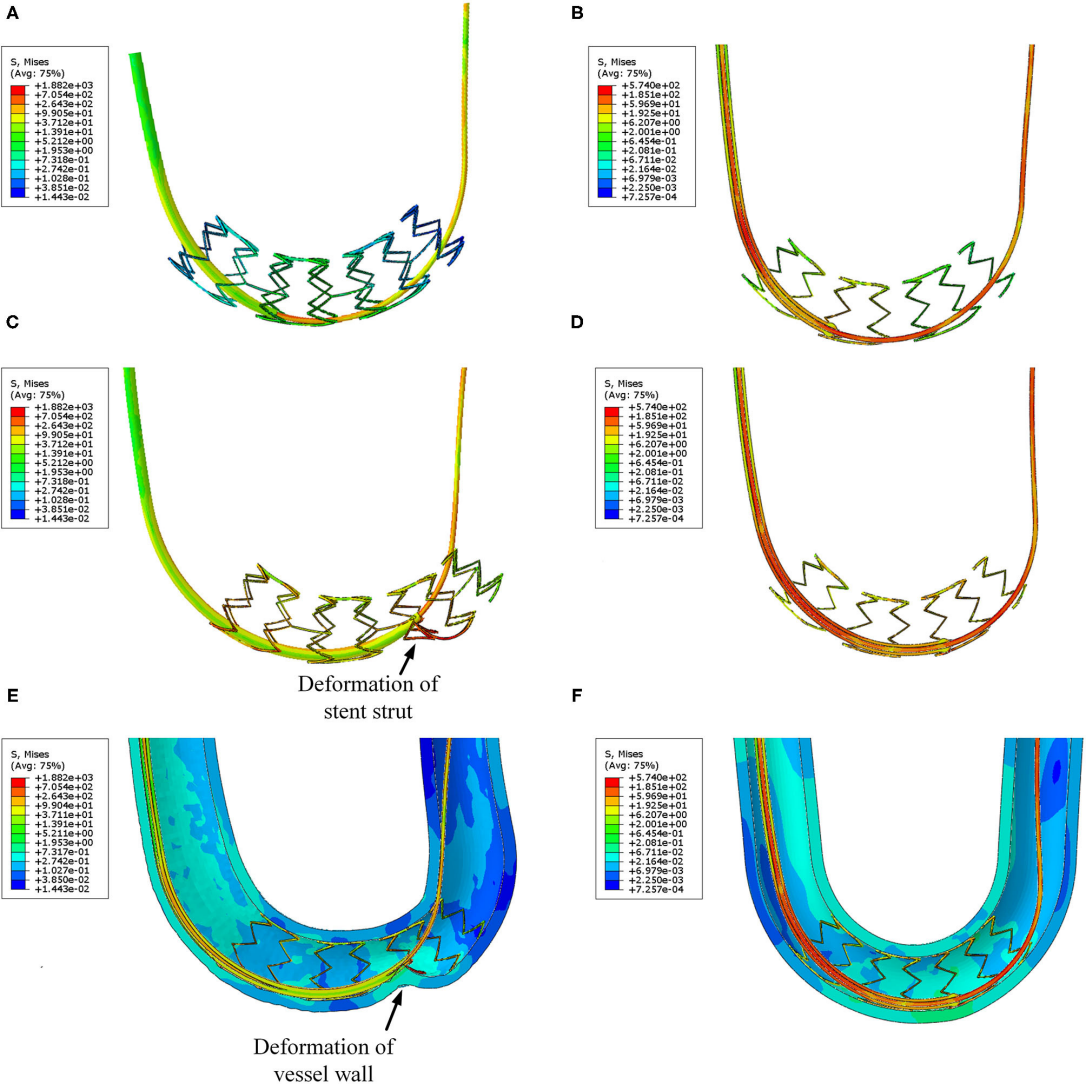
Visualizations of FEA for the post-dilation. **(A)** Stress distribution of the traditional catheter at 0.8 s, **(B)** stress distribution of spherical-tip catheter at 0.8 s, **(C)** stress distribution of traditional catheter at 1.1 s, **(D)** stress distribution of spherical-tip catheter at 1.1 s, **(E)** section view of the traditional catheter model, and **(F)** section view of the spherical-tip catheter model.

The stress distribution of the two catheter models at different time are shown in Figures 11A–D. It can be seen that the spherical-tip catheter can pass smoothly, while the traditional catheter is blocked and causes the stent to deform. This demonstrates the better passability of the spherical-tip catheter in comparison to the traditional one. The tip of the spherical-tip catheter slips over the stent struts when it comes into contact with the stent struts. Contrariwise, the traditional catheter does not pass the stent struts unless the stent is deformed as shown in Figure 11C. The section view of stress distribution of the two catheter models and vessel deformation at 1.1 s are presented in Figures 11E,F. As shown in Figure 11E, the vessels and the stent undergo large deformations together when the catheter tip gets stuck. This likely is the cause of tissue damage in clinical practice.

**Figure 12:**
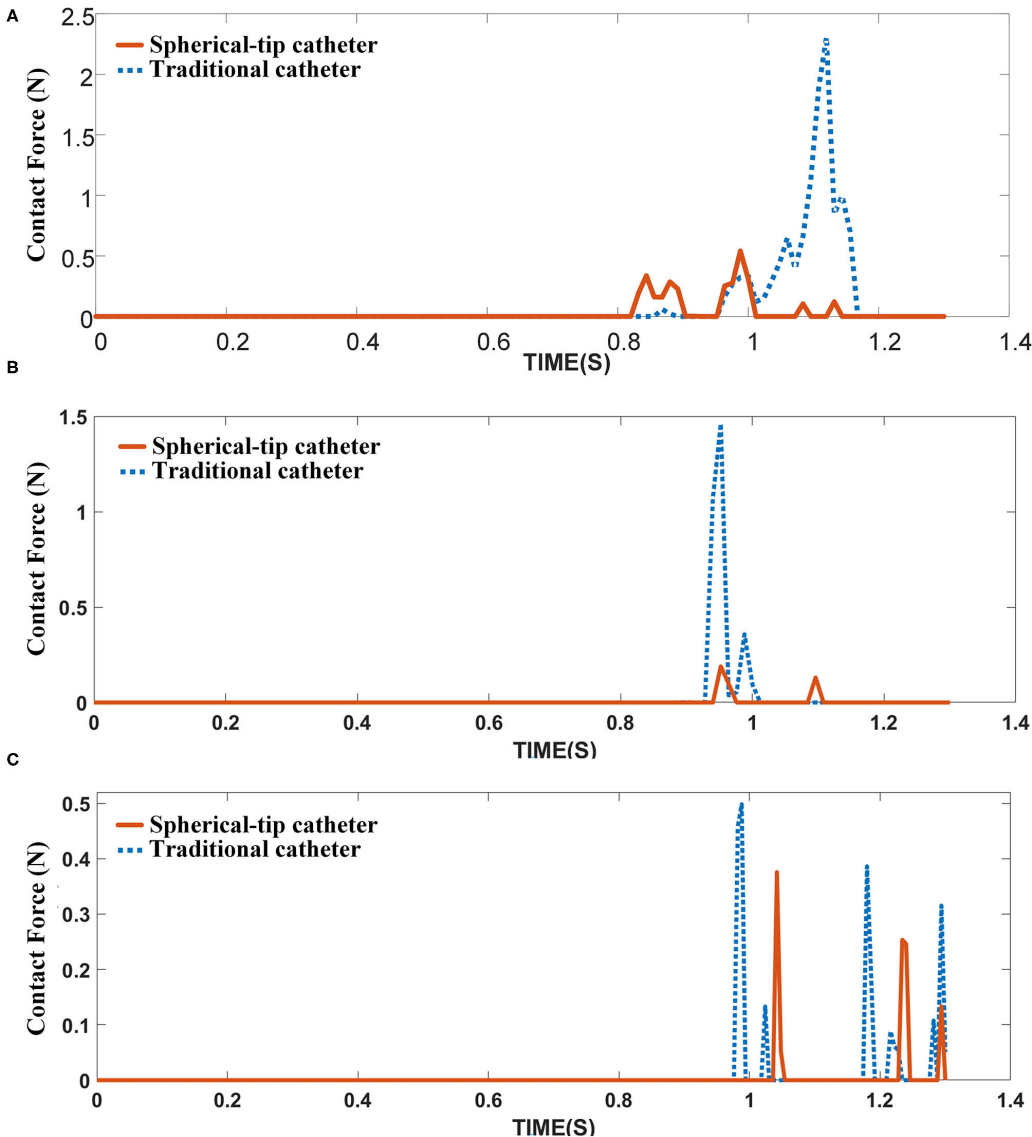
Contact force between catheter tips and the inner surface of the stent under different curved vessels with **(A)** curvature radius of 10 mm, **(B)** curvature radius of 20 mm, and **(C)** curvature radius of 30 mm.

Figure 12A shows the contact force between the catheter tip and stent of two FE models. None of the two catheters touches the stent struts before 0.8 s. Then, the spherical-tip catheter firstly comes into contact with the stent strut. The contact force is discontinuous because there are gaps between the struts. The maximum value of the contact force for the traditional model is 2.3 N, which is much larger than the force (0.54 N) in the spherical-tip catheter model. It indicates that the spherical-tip catheter passes easier through the stent implanted in a curved vessel than the traditional one. The contact force of the traditional catheter model decreases after reaching the highest point because the catheter tip passes through the stent surface after the large deformation of the stent. As shown in Figures 12B,C, the curved vessel models with curvature radius of 20 mm and 30 mm result in the same conclusion as that of the curvature radius of 10 mm.

**Figure 13:**
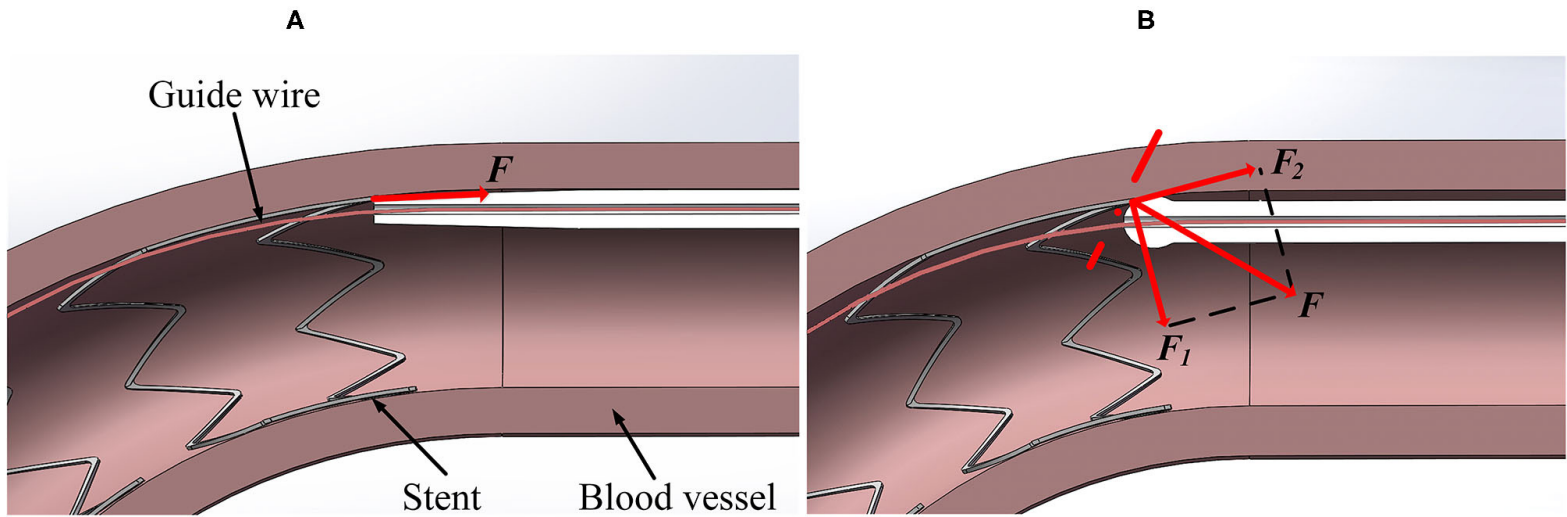
Contact force analysis in different models from section view. **(A)** Traditional catheter model, and **(B)** spherical-tip catheter model.

The contact forces measured in the experiment are in good agreement with the simulation results (see Figures 5, 10), which provides the basis for the FE simulation of catheter-stent-vessel configuration. Meanwhile, it shows that the spherical-tip catheter has less chance of getting blocked during the post-dilation. The FE simulations for the post-dilation reveal that the maximum contact force between the tip of spherical-tip catheter and stent is smaller than that between the traditional catheter tip and stent when advancing catheters. The traditional catheter causes larger deformation of stent and vessel, which may have dangerous consequences, such as tissue damage. When the catheter comes into contact with the stent, the force of the traditional catheter model at the tip is perpendicular to the contact surface, which is opposite to the direction of movement of the catheter, as shown in Figure 13A. The contact force on the tip of the spherical-tip catheter is perpendicular to the spherical contact surface and can be decomposed into two components *F*_1_ and *F*_2_ as shown in Figure 13B. *F*_1_ contributes to the forward movement of the spherical-tip catheter passing across the stent strut. Therefore, it can be inferred that the traditional catheter cannot easily pass through the stent unless the stent is largely deformed. In contrast, the spherical-tip catheter can pass through the stent by virtue of the contribution of the *F*_1_ component.

###  Limitations of the Study

As a pilot study, although the results of the FE modeling might not be completely accurate because of deviations in the material properties, geometrical approximation in the FM mesh, and assumptions in boundary conditions, the combination of FE simulation and experimental measurement to qualitatively evaluate the performance of novel spherical-tip catheter could provide potential implications for clinical trials. In this study, simplified mechanical models were constructed, without the fluid-structure interaction (FSI), and the lubrication effect of blood was neglected. In fact, in the catheter-stent-vessel system, the blood flow can cause the shear-thinning effect, which can significantly affect the wall shear stress (Liu et al., 2018), and the blood can also change the friction on the contact surface (Wagner et al., 2021). Moreover, in the FE model, the blood vessel was shaped as a soft tube with different curvatures under identical inner diameter. In fact, the patient-specific geometry is an important factor in analyzing the passability of catheter, since the 3D curvature and diameter of blood vessel vary in different segments and patients. Therefore, for realistic estimation, the transient FSI, lubrication effect, and patient-specific modeling are noteworthy to be further investigated.

On the other hand, due to the tiny dimension of the catheter tip, it is challenging to measure the deformation of the catheter tip and contact force in the catheter-stent-vessel system. Azarnoush et al. (2019) applied the intravascular optical coherence tomography probe inside the angioplasty balloon and designed some preliminary experiments, to validate the mechanical properties of balloon provided by FE simulation, during the balloon inflation by means of intravascular imaging. This type of intravascular imaging technology provides the potential to quantitatively monitor the interaction between catheter and stent and between catheter and tissue.

## Conclusions

In this study, we present the evaluation on a novel design of balloon post-dilation catheter using an experimentally verified FEA, in which we compared a newly designed spherical-tip catheter with a traditional conical-tip catheter. The simulation results of the novel spherical-tip catheter and traditional one demonstrate that the new type of spherical-tip catheter has a better performance than the traditional one in passing through the stent area within different curved vessels with the curvature radius of 10, 20, and 30 mm. The new catheter has less contact force than that of traditional one and less potential damage to the blood vessel. Also, it is shown that the FM model developed in this study can be a helpful tool in the design of new catheters and an alternative to animal testing, which is always performed before the pre-clinical trials of this catheter as given in the 3Rs principle (replacement, reduction, and refinement of animal studies).

## Data Availability Statement

The raw data supporting the conclusions of this article will be made available by the authors, without undue reservation.

## Author Contributions

LQ and LX: propose the concept and design. LQ, WZ, and WQ: conduct finite element modeling and data analysis. LQ and WZ: draft the manuscript. LX, YH, and FZ: review and revise the manuscript. All authors made contributions to this study and manuscript.

## Conflict of Interest

The authors declare that the research was conducted in the absence of any commercial or financial relationships that could be construed as a potential conflict of interest.

## Publisher's Note

All claims expressed in this article are solely those of the authors and do not necessarily represent those of their affiliated organizations, or those of the publisher, the editors and the reviewers. Any product that may be evaluated in this article, or claim that may be made by its manufacturer, is not guaranteed or endorsed by the publisher.
